# A Day Not Very Far from the Alps, Switzerland

**Published:** 1876-02

**Authors:** 


### A DAY NOT VERY FAR FROM THE ALPS, SWITZERLAND.

*Mr. Editor:*—It has been some time since I have had the pleasure of practicing what one critic calls "the pyrotechnie style of writing," for the old reliable DENTAL REGISTER of the West. Ah! how a western man's heart warms and yearns for the west, when circumstances have placed him far away from this region of freedom, of breadth of thought, of nobleness of nature, of independence of expression—the West. Yankeedom may boast of a little higher cultivation in some art matters perhaps, and of a more precise and rigid style of manners. The South may boast of her graceful and hospital habits, of her boldness and openness of expression, but the West—especially that part about the Ohio and Miami Valleys—is the spot of all the world, where just exactly the right sort of men can be found. If Mr. Editor, or you, Mr. Reader, don't believe this statement—and if you were born and brought up in good old Ohio,—come over to Switzerland or Germany or France or England or Ireland and stay three or four years, and you will see how it is,—you will appreciate what fine fellows you have left behind—you will find yourself looking out for Ohioans wherever you travel, and you will know them on sight. You will come in contact with all the world and be, to a certain degree, dissatisfied with all, and only yourself, your very own self, without restraint or fear of not being understood, when you meet an Ohio boy. He does not open his eyes and stare, if your language is a little florid. He does not "ah! ye-es!" when you tell him your best story. He does not hint at more caution when you express in strong, good Saxon, your feelings of indignation against some evil doer in a high place. Not he, but with a warmth of heart and strength of expression equal to your own, he praises the good, condemns the evil, and speaks the noble, holy truth, without a fear and without a feeling of policy. May age and advancing civilization, and crowding population of Ohio never engender the parsimony, the policy, the shrewdness of older countries in the character of her sons. Let the inflation question and the Roman church question and the Public School question make Ohio the arena, we shall find as much honesty and more outspoken freedom of opinion, with less bitterness of heart here, than anywhere. Your correspondent believes this, Mr. Editor, though the prospect of filling his pockets with gold, led him like many another poor adventurer, to leave this glorious land and come to Switzerland. In Europe, it is believed by many in our profession, lives the great Dental Goose, who offers to lay a golden egg every day in the hand of the dentist who will go to her. The dentist frequently finds that he himself is the best representative of the goose, for practicing in Europe is as near like practicing at home as possible. You can not get something for nothing here any more than at home. If a practice is found ready to hand here in this old country, it is owned by somebody, and is a buyable and salable property, and if you would possess it you must pay dearly for it or let it alone. If you would build up a practice, you must do as you would at home, begin at the first rung of the ladder and mount slowly and carefully, molding yourself a little to suit the character of the people among whom you are living. You can not be the Yankee or the western boy in Europe among your patients and expect to succeed. The man who can the best adapt himself to foreign customs and habits of thought, would succeed first I believe, in gaining the confidence of people who have inherited their habits and customs and trains of thought. Some think they would avoid the fight of competition here, but only the first American dentists of Europe avoided that entirely and they had the more difficult battle of educating people to believe in dentistry at all. Now every city of any importance, boasts of its American dentists, and people do not go to simply the American dentist, but to the one who has been recommended, as in America. In Europe also the character of practices vary as in America, for instance, in a watering place or where the English and American traveler resorts, the American dentists depend largely on the traveling public for patients, while in other quiet places the native practice or practice among the natives is the only dependence, and as the fees of American dentists are high in comparison with the charges of the native dentist, only the cream of intelligence and of society are the patients of the former. He can not expect the great middle class as patients, therefore as you can see the cream of one city of ordinary size is not quite sufficient for an ambitious dentist and he must expect surrounding cities to send him a little of their cream. If we could get the milk as well—oh well! we'd have too much to do at fees that would not pay. A native dentist of the best class opens his office at day light and receives patients till late in the evening. He is rushed and hurried all day, he must talk a great deal and keep his operating room full, and get a few francs for his operations and nothing for most of his advice and talk. Yet he will become rich if he lives long enough and saves his francs. The American dentist on the contrary, while he may work as hard and break himself down in health, works with more system and with better results per hour. If you will allow, I will give one day of an American dentist in Switzerland—in Basel if you please. First know, that in most offices here all the modern improvements in chairs and machinery are to be found, and that offices and laboratories are fitted up as is usual in modern American offices at home. Now let us begin our day. Eight o'clock a. m, the dentist enters his office and receives the correspondence and telegrams, asking in various languages for appointments. These he proceeds to arrange and answer sending his card with the time, day and hour of the appointment, which he enters in his diary. At 8½ the first patient is ushered into the parlor—Madame Blank and little daughter—the little daughter twelve years old is the patient, teeth of the ordinary character found here; white, chalky and extremely liable and subject to caries. The six year molars have been filled with gold in their crown fissures, to-day we have to fill the approximal cavities of the superior incisors. There are four nice little cavities, close to the necks of the teeth, spaces had been made by the cotton wedges for three days. Now wooden wedges are driven and the almost round carious cavities are well exposed, small excavators and the fine burs of White's engine are used and the cavities prepared. The rubber dam will not be used in this case, but the fold of bibulous paper under the lip and the small linen napkin held by the finger under the teeth against the palate. Williams' B. cylinders are just the things here and in three or four minutes the first cavity is filled with three of the cylinders, two B. and a narrow strip of foil wedged in as the key. The surface is malleted with the new White's engine mallet—a glorious good thing by the way. By 10½ o'clock the four small cavities are filled, filed and polished, and the patient dismissed for three months. At 10½ Mr. Blank comes by appointment with a bad second left bicuspid, posterior cavity to be filled. Authur's disks are used and enough space made to work comfortably. Notice is taken of the articulation of this tooth with the lower. A groove is cut in the cervical wall, the cavity is united with the fisure in the crown, when prepared. Williams' three A. six cylinders are used as the foundation at the cervical wall, unannealed. Upon these, lighty pressed into position, the building process is begun, with the smaller cylinders A. 1., annealed, and as we are trying Leslie's crystaline gold we use this too, employing the engine mallet from the bottom up, but trying not to over mallet, as we agree with the last named gentleman entirely in his written and spoken statement about the properties of all gold. The coffer dam was used in this case and the gold brought over into the fissure of the crown. By 12¼ o'clock we have finished this; our back is broken and the patient looks like a damp cloth. These are the positions when we can not learn to sit while filling but stand over our low chair with the mirror in the left hand, working at what we see in the mirror. Now the morning is over, let's record our work. Four fillings at 20 francs equal 80 frances, for two hours. One compound filling 1¾ hours, 60 francs. 140 francs for the morning or about $28 for some rapid work and hard work. Now comes a rest and lunch, and a joke or two with the babies, from 1 to 2 o'clock is the doctor's consultation hour, when he is always free to be consulted and no appointments are made for this hour. One or two persons come for examination or for wedges or for treatment, for treatment and examinations a fee of 5 francs is charged. At 2 begins the afternoon work. A patient from a neighboring city comes by appointment with her three boys. She comes regularly every six months and her boys have good solid, ugly teeth, ugly because they can't be persuaded to clean them well more than once a week. The three take turn about in examination and as, (six months ago they were pretty generally finished off,) we find but two cavities of decay for the three and these are in the sutures of new inferior second molars. These are gone for—with the chisels and engine, and mostly filled with cylenders, finishing with Pack's pellets malleted on. The corundum wheel and finishing bur lets this boy out of the chair at 3 o'clock, after about 40 minutes work. The other twenty being occupied in examination of the others. Now the boys take turn in having their teeth scaled and polished, and a few minutes before 4 o'clock, the record is made. Mad. ——— for son Karl two gold at 20 equal 40 francs; attention to teeth of sons, 20 francs; total 60 francs; total per day, 200 francs. Oh! here is a telegram that gives pleasure,—"Meet me at Hotel des Jrois Rois at 8 o'clock, Bogue"—and we leave the office for a walk with our Frau or a game at billiards, or a smoke of a good cigar and a glass of beer in a cafe. At 8 o'clock we have one of the few pleasures of practice here, we meet and talk with an American dentist—Dr. Bogue, of New York, does come as per telegram—and we sit and have a little supper on the banks of the Rhine in a cafe garden and then we feel more than ever home sick and wish we had the conflict of dental practice at home instead of the jog-trot, dull, easy, slow, sleepy life of Basel, Switzerland. For a man gets rusty and dusty over here, he gets old in this old country and as he gazes at the dregs in his beer mug, he thinks the froth and sparkle were left in America, and as he sees the white ashes of his Havana and the curly smoke gradually leaving in his hand nothing but an old stump of a cigar, he feels that he is very much like the old stump. The American Dental Society of Europe is just here remembered and the dentist's eye brightens and the future looks brighter, for this society is the blessing to the expatriated practitioner of dentistry in Europe, and your correspondent is only sorry that he was not still more abusive to "Vagrant, in Johnston's Miscellany" who did not criticise, but cast slings at our society, even if he has learned since that Vagrant only likes to cover up his wicked slings by this name instead of coming out as an Ohio man would and saying that he is a professor of mechanical dentistry in London. Yours truly, C. M. Wright,

Basel, Switzerland.

P. S. I won't tell of dark, rainy days when none of your patients come, and if they do, you can't see to operate and lose your time. Nor will I tell of the hot days or the cold days, or the vacation times when you have no appointments and can attend to odds and ends of undone things, for if I should, I might discourage some dentists from coming to Europe for a pocket full of money—and we want dentists to come for company.

